# Editorial: Novel computational fluid dynamics methods for diagnosis, monitoring, prediction, and personalized treatment for cardiovascular disease and cancer metastasis

**DOI:** 10.3389/fbioe.2024.1491950

**Published:** 2024-10-15

**Authors:** Zahra Keshavarz Motamed, Nima Maftoon, Lakshmi Prasad Dasi, John F. LaDisa

**Affiliations:** ^1^ Department of Mechanical Engineering, McMaster University, Hamilton, ON, Canada; ^2^ Department of Systems Design Engineering, University of Waterloo, Waterloo, ON, Canada; ^3^ Wallace H. Coulter Department of Biomedical Engineering, Georgia Institute of Technology, Atlanta, GA, United States; ^4^ Department of Biomedical Engineering, Medical College of Wisconsin and Marquette University, WI and Department of Pediatrics, Section of Cardiology, Herma Heart Institute, Children’s Wisconsin and the Medical College of Wisconsin, Milwaukee, WI, United States

**Keywords:** hemodynamics, simulation, computers, multiscale modeling and computation, wall shear stress (WSS), patient-specific 3-D model

This Research Topic features recent advances in computational hemodynamic methods for diagnosis, prediction, and personalized treatment planning applicable to cardiovascular disease and cancer metastasis. The Research Topic of articles describes novel tools, therapeutic and process improvement methods, and studies exploiting the capability of computational tools to provide quantitative information about hemodynamics and interactions with cells and tissues beyond conventional clinical possibilities.

For example, Wang et al. present exciting results showing patient specific steady computational fluid dynamics (CFD) simulations following virtual splenectomy that may predict the likelihood of thrombosis (Wang et al.). Preoperative imaging data were used for model creation from portal hypertensive patients who underwent splenectomy. Results show the area of low wall shear stress (WSS; defined as <20% of the patient-specific average) can predict post-splenectomy thrombosis with an area under the receiver operating curve of 0.75. Diameter of the splenic vein was correlated with WSS results, which was further validated in another small group of patients. Extension of this work may influence future postoperative management and prophylaxis protocols in this patient population.

Assi et al. conducted patient-specific CFD with ultimate application to the prediction of thrombosis using simulations with physiologic boundary conditions to characterize hemodynamics from iliac vein compression syndrome patients relative to controls (Assi et al.). Their results from this understudied population showed increased shear rate in the left *versus* right common iliac vein of patients (resulting in a higher ratio), and relative to controls. This work serves as an exciting foundation for shear rate and the shear rate ratio between contralateral iliac veins to serve as potential measures of thrombosis risk in iliac vein compression syndrome patients.

Citing alterations in endothelial cell transport based on local WSS distributions, Rahmati and Maftoon, 2024 used an idealized vessel with variable curvature to simulate the role of WSS from tortuosity on interactions between circulating tumor cells and the vessel wall (Rahmati and Maftoon). Simulation methods included fluid-structure and receptor-ligand interactions for circulating tumor cell (i.e., deformable body) adhesion within a simulated plasma environment via an immersed boundary approach. Curvature, asymmetrical flow patterns and associated WSS alterations established local adhesion dynamics. Expansion of such methods may ultimately prove useful in assessing the likelihood of metastasis.

Colombo et al. describe a workflow using *in vitro* and *in silico* approaches to further study hemodynamics in the microvasculature and build from prior papers associating WSS alterations with coronary microvascular disease (Colombo et al.). In short, the workflow created an idealized microvasculature representation based on Murray’s law, which was then printed using 3D molds and ultimately seeded with human embryonic kidney cells to study their response to varying degrees of microvascular disease (i.e., impaired flow to several microvasculature model outlets).

Lopez-Santana et al. employed CFD simulations with multiple inlets, Windkessel outlet boundary conditions, and geometry from a healthy patient to characterize aortic flow patterns resulting from different geometric parameters related to the anastomosis of a left ventricular assist device outflow graft (Lopez-Santana et al.). Specifically the authors vary the angle of insertion, distance along the ascending aorta from the anatomic ventriculoarterial junction, and cardinal position around the aorta (coronal to sagittal) as parameters, and characterize results in terms of WSS, pressure, vorticity and turbulent kinetic energy.

Kim et al. quantified differences in left atrial morphology and associated readouts related to the potential for stroke (i.e., velocity and stasis) using models created from phase-contrast as compared to contrast-enhanced magnetic resonance angiography (Kim et al.). Results were promising, suggesting their segmentation and registration workflow allows for the use of contrast enhanced imaging data for 4D flow analysis with minimal impact on the readouts assessed.

Alamir et al. applied a U-net based deep learning approach to estimate WSS in atherosclerotic coronary arteries without associated branches using seven spatial features (e.g., Cartesian coordinates for arterial borders and centerline) and velocity, together with geometrical features (e.g., curvature vector and distance along model centerline) aimed at improving predictive power (Alamir et al.). Normalized mean absolute error relative to WSS results from steady CFD simulations was ∼6%, but results were obtained in just 0.35 s.

Novel computational hemodynamic analysis tools may enable the next-generation of diagnostic, predictive, and treatment planning tools. Of note, a commonality in the work featured includes consideration of inter-subject variability along with circulatory anatomy and pathophysiology to provide personalized diagnosis, prediction, and treatment. This critical component may ultimately lead to clinical utility through extension of the foundational work reviewed above and found within the details of the current Research Topic.

